# An efficient polynomial-based verifiable computation scheme on multi-source outsourced data

**DOI:** 10.1038/s41598-024-53267-x

**Published:** 2024-04-12

**Authors:** Yiran Zhang, Huizheng Geng, Li Su, Shen He, Li Lu

**Affiliations:** China Mobile Research Institute, Beijing, 100053 China

**Keywords:** Computer science, Information technology

## Abstract

With the development of cloud computing, users are more inclined to outsource complex computing tasks to cloud servers with strong computing capacity, and the cloud returns the final calculation results. However, the cloud is not completely trustworthy, which may leak the data of user and even return incorrect calculations on purpose. Therefore, it is important to verify the results of computing tasks without revealing the privacy of the users. Among all the computing tasks, the polynomial calculation is widely used in information security, linear algebra, signal processing and other fields. Most existing polynomial-based verifiable computation schemes require that the input of the polynomial function must come from a single data source, which means that the data must be signed by a single user. However, the input of the polynomial may come from multiple users in the practical application. In order to solve this problem, the researchers have proposed some schemes for multi-source outsourced data, but these schemes have the common problem of low efficiency. To improve the efficiency, this paper proposes an efficient polynomial-based verifiable computation scheme on multi-source outsourced data. We optimize the polynomials using Horner’s method to increase the speed of verification, in which the addition gate and the multiplication gate can be interleaved to represent the polynomial function. In order to adapt to this structure, we design the corresponding homomorphic verification tag, so that the input of the polynomial can come from multiple data sources. We prove the correctness and rationality of the scheme, and carry out numerical analysis and evaluation research to verify the efficiency of the scheme. The experimental indicate that data contributors can sign 1000 new data in merely 2 s, while the verification of a delegated polynomial function with a power of 100 requires only 18 ms. These results confirm that the proposed scheme is better than the existing scheme.

## Introduction

Cloud computing technology has become an indispensable tool in the Internet era, and it has gradually penetrated into all aspects of daily life. Users with weak computing capabilities tend to outsource complex computing tasks to cloud servers with powerful computing and storage capabilities, thus reducing the complexity of local computing^1^. However, cloud servers are not completely trustworthy. The cloud servers have the potential to leak user data or intentionally return incorrect calculation result. Therefore, it is of practical significance to verify the calculation results of outsourcing services without disclosing user privacy^2,3^.

Verifiable computation (VC, verifiable computation) solves the above problem. The user sends the function and the input data of the function to the cloud server, and the cloud server returns the calculated result and the proof of the result. Users can verify the correctness of the calculation results, and the computational complexity of this process is much smaller than that of directly calculating functions. Verifiable computation is generally divided into two categories: (1) verifiable computation of general functions, which is suitable for the computation of any function^4,5^; (2) verifiable computation of special functions, such as modular exponential operation^6,7^, polynomial calculation^8^, attribute-based decryption operation^9^, etc. Among them, the polynomial-based verifiable computation is widely used in information security, linear algebra, signal processing and other fields, so it has attracted wide attention.

### Motivation and contribution

The researchers have proposed some verifiable computation based on polynomial schemes^10–35^. Benabbas et al.^10^ put forward a polynomial outsourcing computing scheme for the first time. The scheme requires that the input of the polynomial must come from a single data source, which means that the data must be signed by a single user. However, the input of the polynomial may come from multiple users in the practical application. In order to solve this problem, the scheme^11^ is proposed to design an outsourced polynomial computation program based on the idea of homomorphic verifiable computation tags, and make the scheme support multiple data sources. However, the scheme requires that all multiplication gates must be executed before the addition gate when generating verification tag, which greatly limits the speed of generating the verification tag and leads to low efficiency. Want et al.^12^ improved the scheme^11^, but it only improved the security of signatures and do not consider the efficiency degradation caused by he design of verification tag. In particular, when the order of the polynomial function is relatively high and the data of the same user is calculated many times, the correctness verification of the result will be extremely slow.

From the above references, there are two problems with the proposed scheme. First, the existing scheme requires that the input of the polynomial must come from a single data source; Secondly, the design of verification tags may cause a decrease in efficiency, especially when the polynomial function is relatively complex, so that the result correctness verification process will be extremely slow, and even affect the use of data. Therefore, we define two key requirements for efficient verifiable computation schemes on multi-source outsourced data. (1) Efficient. The scheme should ensure that the verification can be completed quickly. (2) Support for multiple data sources. The input of the polynomial can come from multiple independent data sources, which means that the data from different data sources can be signed with different private keys.

To address these issues, we propose a new and efficient polynomial-based verifiable computation scheme on multi-source outsourced data, which has the characteristics of efficient and supporting multiple data sources. We optimize the polynomials using Horner’s Method to increase the speed of verification, in which the addition gate and the multiplication gate can be interleaved to represent the polynomial function. In order to adapt to this structure, we design the corresponding verification tag, which is additive homomorphism and multiplicative homomorphic, so as to suit for all types of polynomials. We have verified the correctness and soundness of the scheme based on Computational Diffie-Hellman(CDH) Assumption. The experimental prove the efficiency of the scheme.

The main contributions of this paper can be summarized as follows:

1. We design for the first time an efficient polynomial-based verifiable computation scheme on multi-source outsourced data, which has the characteristics of efficient and supporting multiple data sources. For multi-source outsourcing systems, the cloud server can perform polynomial functions to obtain the calculation results and generate proof information, which can be used by third parties to verify the correctness of the calculation results without knowing the input.
2. In order to solve the problem of single data source, this paper designs a homomorphic verification tag structure that supports multiple data sources. As the polynomial function is executed gate by gate, we use the key management center to convert the signatures signed by different user into the verification tag with the unified public and private keys, so that the input of the polynomial can come from multiple data sources.
3. In order to solve the problem of low efficiency, we optimize the polynomials using Horner’s Method, and the generation of corresponding verification tag can be generated with the cross-operator of multiplication gate and addition gate, so as to improve the verification speed.

## Related work

Gennaro et al.^13^ combined outsourced computation and verification technology to propose the concept of verifiable computation for the first time. It constructed an outsourced scheme of verifiable computation by using obfuscated circuits and full homomorphic encryption, which can ensure the privacy of input and output. However, this scheme can only do private verification. Benabbas et al.^10^ proposed a polynomial outsourcing computing scheme with Chosen Plaintext Attack (CPA) security, which solved the problem left by Gennaro et al.^13^ The scheme used addition homomorphic encryption algorithm to ensure the privacy of the polynomial, but could not guarantee the privacy of inputs and realize public verification. Zhang et al.^14^ constructed a univariate polynomial outsourcing calculation scheme by using multilinear mapping and homomorphic encryption algorithm. This scheme can ensure the privacy of input, and its extension scheme can ensure the privacy of function, but it can only achieve private verification. Papamanthou et al.^15^ proposed a verifiable outsourcing computation scheme for dynamic polynomials that allows incremental updating of the coefficients. Fiore et al.^16^ proposed a verifiable polynomial outsourcing computation scheme with adaptive security, but this scheme can only guarantee the privacy of the function. Zhang et al.^18^ improved the efficiency of IOT cross-chain computing by outsourcing polynomials to the blockchain, and they proposed an efficient and verifiable polynomial cross-chain outsourcing computing scheme for verifying the correctness of the results of calculations on the blockchain, but the practicality of the scheme is modest.

Other researchers have proposed verifiable computation schemes based on homomorphic signatures^19–30^. Barbosa et al.^19^ put forward the Delegatable Homomorphic Encryption (DHE) cryptographic primitive, and give a method on how to use DHE to construct a verifiable computation scheme. In recent years, Guo et al.^20^ has developed a lightweight verifiable blind decryption technique based on a linear homomorphic encryption scheme to verify the correctness of the final result. Boneh and Freeman^21^ proposed the implementation of homomorphic signatures based on polynomials of constant degree, but this scheme can only be applied to the verifiable computation of polynomials of constant degree. Fiore and Gennaro^22^ proposed a publicly verifiable secure outsourcing protocol for polynomial and matrix multiplication evaluation. However, this scenario does not support multiple data contributors. Song et al.^23^ proposed a verifiable computation scheme that supports multiple data sources. It is based on the verifying data structure of the Homomorphic Verifiable Computation Tags, which is only an additive homomorphism. However, polynomials not only have addition operations, but also multiplication operations, so the scheme is not functional enough to support polynomial computation. Further, song et al.^11^ proposed a verifiable polynomial computation scheme that supports multiple data sources. When two inputs are signed by different keys from different data contributors, it is difficult to have a uniform validation data structure to support addition and multiplication. To solve this problem, the idea is to place all addition gates behind product gates to represent delegated polynomial functions. Then, based on this structure, they further designed the first-level verification label and the second-level verification label. By utilizing these designs, the server is able to output homomorphic validation labels for each gate even if the validation labels for the two inputs are signed by different keys. However, it is clear that executing the addition gate after the multiplication gate will affect the speed of the verification label generation, especially if the input with a data source is evaluated multiple times. Although Want et al.^12^ improved the scheme^11^, it only improved the security of signatures and did not pay attention to the inefficiency. Although there are few solutions solve the problem of correctness verification of polynomial calculation with multi-sources, they do not pay attention to the efficiency reduction caused by the designing of scheme.

## Preliminaries

### Arithmetic circuit

#### Definition

 Arithmetic circuits^36^ on fields F and variable sets $X={x_1,\ldots ,x_n}$ have two kinds of gates: multiplication gate ’$\times$’ and addition gate ’+’. Every gate marked with the ’x’ is called the product gate, and every gate marked with the ’+’ is called the sum gate.

The arithmetic circuit computes polynomial functions, where the product gate computes the product of polynomials on its input wire, and the summation gate computes the sum of polynomials on its input wire. In this paper, the cloud server performs gate-to-gate processing of polynomials based on arithmetic circuits.

### Bilinear mapping

Bilinear mapping refers to the linear mapping relationship between two cyclic groups^37^. We define the mapping $e:G_1\times G_1\rightarrow G_2$ as a bilinear mapping, where $G_1$ and $G_2$ are multiplicative cyclic group of order *p*, and *g*, *h* are two generators of the group $G_1$. It satisfies the following properties:

1. Bilinear: for $a,b\in Z_p, g^a,g^b,h^a,h^b\in G_1$, then $e\left( g^a,g^b\right) =e{(g,h)}^{ab}$ can be calculated.
2. Non-degenerate: $$e\left( g,g\right) \ne 1$$.
3. Computability: For any $g,h\in G_1$, there are effective algorithms that can calculate $e\left( g,h\right)$.

Computational Diffie-Hellman (CDH) Assumption: For *x*, $y\in Z_p$, there are *g*, $g^x$, $g^y\in G_1$, then it is difficult to compute $g^{xy}$.

### Horner’s method

Horner’s method^38^ is a polynomial evaluation method with a single data source, aiming to simplify polynomial calculation. It transfers a polynomial of degree *n* to *n* linear functions of degree one, and it can be represented as an equation:

1 $$\begin{aligned} f(x)&=a_0+a_1\ x+a_2\ x^2+\ldots +a_n\ x^n\\&=a_0+x(a_1+x(a_2+\ldots +x(a_(n-1)+xa_n\ )))\\ \end{aligned}$$

For a polynomial $f\left( x\right)$ with a single data source, it only needs to perform *n* multiplications and *n* additions, with a time complexity of $\mathcal {O}\left( n\right)$. Compared to normal evaluation, which requires $n(n+1)/2$ multiplications and *n* additions, resulting in a time complexity of $\mathcal {O}\left( n^2\right)$, Horner’s Method is a faster and better way to compute higher-order polynomials.

## Problem statement

### System model

There are three entities in the system model of this scheme: the cloud, the users, and the key management center (KMC).

#### Cloud

It provides storage services for users and computes polynomial functions on outsourced data. And it generates the proof message to verify the correctness of the calculation results. It’s not entirely trustworthy.

#### Users

They outsource their data to the cloud. And they also upload signature data to verify the correctness of the polynomial calculation results. We assume that there are $n(n\ge 1)$ users. They are completely trustworthy.

#### Key management center

It assigns keys to users and helps the cloud generate verification information. After the polynomial function is computed, it verifies the correctness of the result based on the proof information. It’s completely trustworthy.

**Figure 1:**
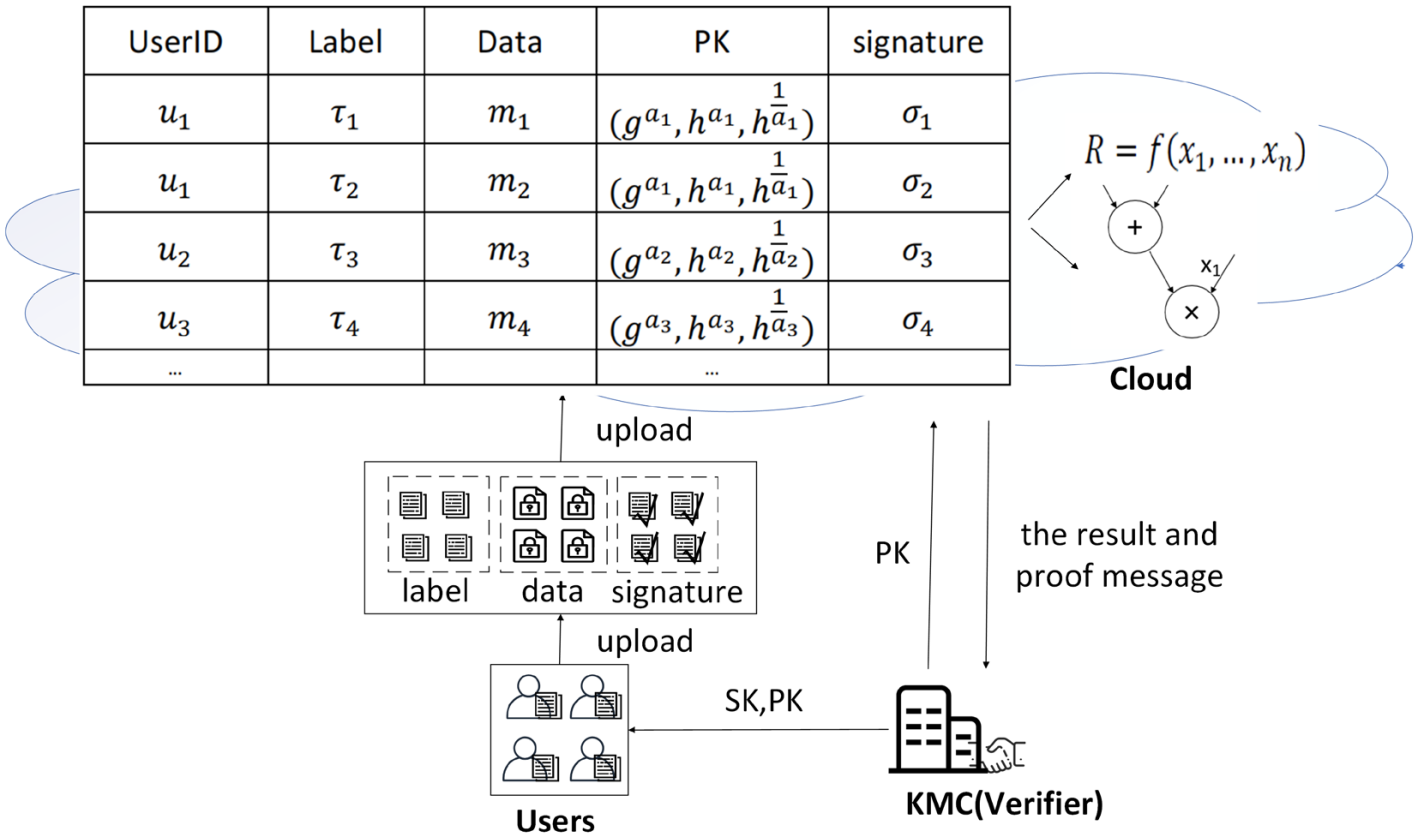
System model.

Figure 1 represents the system model of “An efficient polynomial-based verifiable computation scheme on multi-source outsourced data”. In a multi-source data verifiable computing system, there are *n* user $u_i(1\le i\le n)$. Each user $u_i$ holds their own public key and private key. The user $u_i$ generates signature by signing the data with the private key, then the user $u_i$ uploads signature and data to the cloud. The cloud calculates the polynomial to obtain the calculation result, and it also outputs the proof information. The cloud sends the result and the proof information to KMC. KMC helps the user verify the correctness of the result using the proof information.

### Threat model

The cloud is not completely trustworthy. It may cause misbehavior due to monetary reasons, hacking or system failure. In practical applications, there is a risk that the cloud server may produce incorrect calculation results for users without actually performing the computation. The cloud server may even deliberately provide incorrect calculations. Consider, for instance, a scenario where 5000 users (acting as urban pollution data collection points) in 5000 cities gather information on air pollution from various locations. These users upload their air pollution data to the cloud daily, and request that the cloud calculate the average air pollution based on data from multiple locations. However, the cloud server may perform the calculation using only a subset of the data instead of the entire dataset, leading to erroneous results. Even worse, the cloud server may not perform the computation and return historical data directly or generate random numerical values for the user. Therefore, the work is primarily motivated by the need to provide a verifiable polynomial evaluation scheme. This scheme allows users to verify that the cloud server has correctly executed the entrusted polynomial function. The security threats of this scheme are as follow:

1. Data corruption: The adversary may compromise data during the data is uploaded to the cloud. The corrupted data used as input for polynomial may result in incorrect results.
2. Incorrect results: For monetary reasons, the cloud may not be able to fully execute the entrusted polynomial, or output the result randomly to save computing resources.
3. Forgery attacks: The adversary may forge the signature and proof information on purpose, in order to trick the user into passing the correctness verification.

### Security goal

The security goal of the proposed scheme is twofold: correctness and soundness.

1. Correctness: the cloud performed the polynomial correctly, then the corresponding proof information can pass the correctness check of result, that is, there are no false negatives.
2. Soundness: the verification information corresponding to the wrong result must be detected and fail the correctness check, that is, there are no false positives.

## Efficient polynomial-based verifiable computation scheme on multi-source outsourced data

### Notations in this section

Table 1 shows some important notations.

**Table 1:** Notations.

Notations	Description
$G_1,G_2$	The group of the same prime order *p*
*g*, *h*	The generator of $G_1$
*e*	Bilinear mapping $e:G_1\times G_1\rightarrow G_2$
*H*	One-way hash function $H:\left\{ 0,1\right\} ^*\rightarrow Z_p$
*sk*	Private key $sk=a$
*pk*	Public key $pk=(g^a,h^a,h^\frac{1}{a})$
$\tau$	The label of the outsourced data
$t_\tau$	$t_\tau =H(\tau )$
*m*	The outsourced data *m*
$\sigma _m$	The signature $\sigma _m=\left( r,s\right)$ of data *m*
$\delta$	The verification tag $\delta (pk,\sigma )$
*P*	The proof message $P=\delta _R(pk,\sigma )$ which is the final verification tag for the result of polynomial function

### Overview

The system executes the algorithm SetUp() to initialize the system parameters. KMC performs the algorithm KeyGen() to obtain the public keys and private keys. The users execute algorithm Sign() for signing the data, and the data and the corresponding signatures are outsourced to cloud. The cloud computes the polynomial function to obtain calculation result, and the cloud executes the algorithm GateVal() to obtain the verification tag. As the circuit is executed gate by gate, the verification tag of the last gate is output as the final proof information. The cloud executes the algorithm ProofCre() which sends the proof information to the KMC. KMC executes the algorithm VerifyProof() which verifies the correctness of the final calculation result. If the output is True, it shows the result is correct; if the output is False, it shows that the result is incorrect.

### The proposed scheme

#### ${SetUp\left( 1^\lambda \right) \rightarrow (e,p,G_1,G_2,h,H)}$

The algorithm is executed by the cloud to generate system parameters. The input is the security parameter $\lambda$, and the output is the security parameter of system ${{e,p,G_1,G_2,h,H}}$.

Suppose that $G_1,G_2$ are two p-order prime groups, *g*, *h* are generators of $G_1$, *e* is a bilinear mapping $e:G_1\times G_1\rightarrow G_2$. $H:\left\{ 0,1\right\} ^*\rightarrow Z_p$ is a hash function that maps any string to an element in $Z_p$.

#### $KeyGen\left( 1^k\right) \rightarrow (pk,sk)$

The algorithm is executed by the key management center to generate public keys and private keys. The input is the security parameter *k*, and the output is public key *pk* and private key *sk*.

KMC randomly selects $a^*\in Z_p$ as the conversion private key. When a new user joins the system, KMC randomly selects a random number $a\in Z_p$ as the private key *sk* of the user, generates and stores $a\prime$ satisfying $a*a\prime =a^*$, and outputs public key $pk=(g^a,h^a,h^\frac{1}{a})$. KMC sends the *sk* and *pk* to the user, then sends the *pk* to the cloud.

#### $\varvec{Sign\left( m,sk\right) \rightarrow \sigma _m}$

The algorithm is executed by the user to sign the data. The input is the outsourced data *m* and private key *sk*, and the output is the signature $\sigma _m$.

We set the label $\tau$, which is selected by the user to express the physical implication for the data *m*. And the label $\tau$ is public. The user computes $t_\tau =H\left( \tau \right)$, chooses *k* at random, computes $r=h^k,s=h^{a\left( t_\tau +m+k\right) }\ mod\ p$, where *a* is the private key. Then it generates signature $\sigma _m=(r=h^k,s=h^{a\left( t_\tau +m+k\right) }\ mod\ p)$. Finally, the user uploads data *m*, the label $\tau$, and signature $\sigma _m=(r,s)$ to the cloud.

After receiving data *m* and the corresponding signature $\sigma _m$, the cloud verifies the signature as shown in Eq. (2), where $pk^{(1)}=g^a$. If the verification is successful, the cloud stores the data *m*, the label $\tau$, signature $\sigma _m$, otherwise, the cloud outputs $\bot$.

2 $$\begin{aligned} e\left( g,s\right)&=e\left( g^a,r*h^{t_\tau +m}\right) \\&=e(pk^{\left( 1\right) },r{*h}^{t_\tau +m})\\ \end{aligned}$$

Table 2 shows the userID, labels, data, public keys, and signatures of the users. Each user has a public key, such as the public key corresponding to $u_i$ is represented as $(g^{a_i},h^{a_i},h^{{\frac{1}{a}}_i})(1\le i\le n)$. A user can upload multiple data. For example, data $m_1$ and $m_2$ are uploaded by $u_1$.

**Table 2:** The cloud stores label, data, public key, signature of the user with userid.

UserID	Label	Data	Public key	Signature
$u_1$	$\tau _1$	$m_1$	$(g^{a_1},h^{a_1},h^{{\frac{1}{a}}_1})$	$\sigma _1$
$u_1$	$\tau _2$	$m_2$	$(g^{a_1},h^{a_1},h^{{\frac{1}{a}}_1})$	$\sigma _2$
$u_2$	$\tau _3$	$m_3$	$(g^{a_2},h^{a_2},h^{{\frac{1}{a}}_2})$	$\sigma _3$
$u_3$	$\tau _4$	$m_4$	$(g^{a_3},h^{a_3},h^{{\frac{1}{a}}_3})$	$\sigma _4$
$\ldots$	$\ldots$	$\ldots$	$\ldots$	$\ldots$

#### $\varvec{GateVal()\rightarrow \delta }$

The algorithm is executed by the cloud to generate verification tags. The inputs of a gate could be the original outsourced data, the constant $c\in Z_p$, or the output of the previous gate. The output is verification tag $\delta$. As the circuit is executed gate by gate, the verification tag of the previous gate output is used as the input for the next gate.

**Figure 2:**
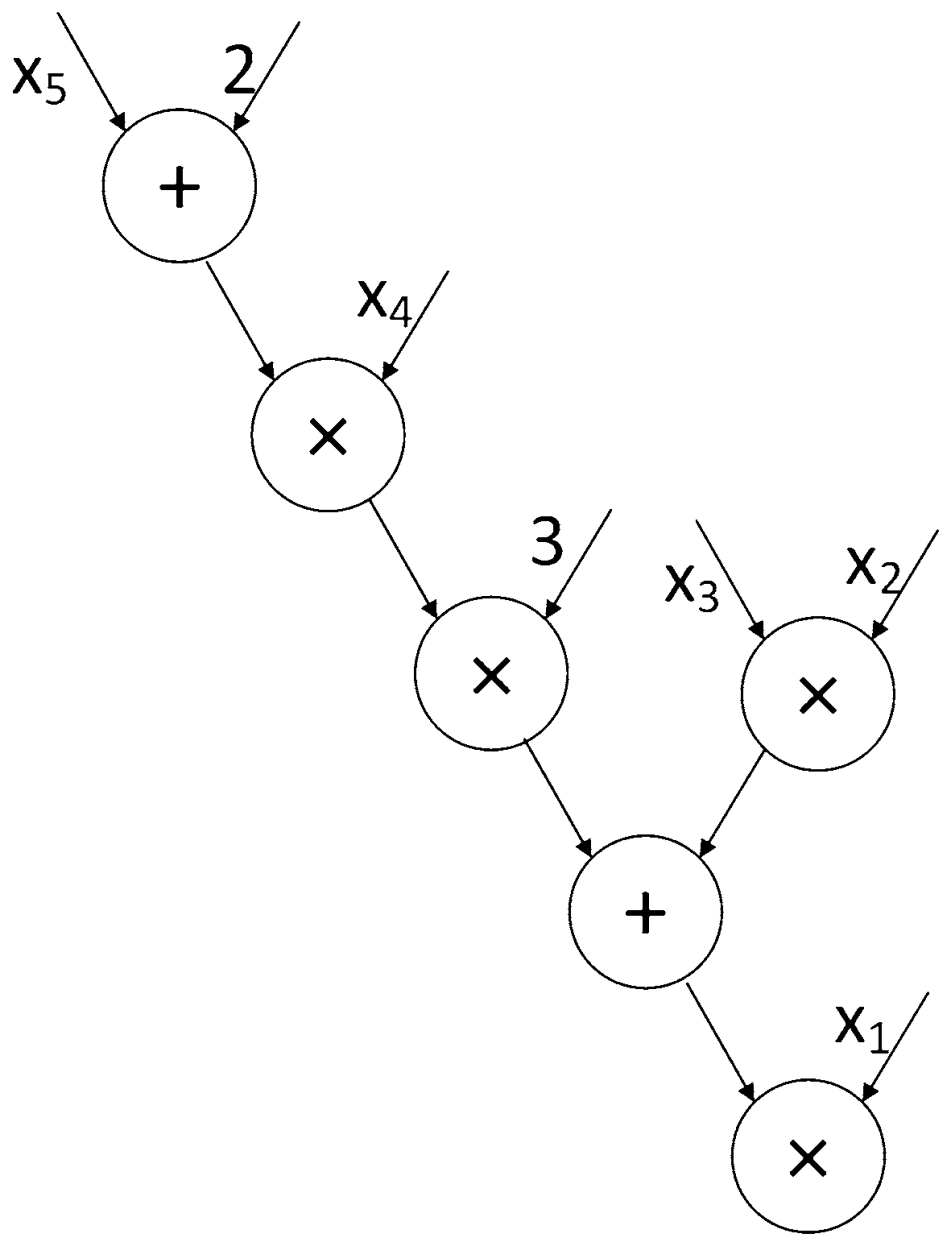
Polynomial functions are represented by arithmetic circuits. For example, $f=x_1x_2x_3+3x_1x_4x_5+6{x_1x}_4=x_1(x_2x_3+3x_4\left( x_5+2\right) )$.

Let $f\left( x_1,\ldots ,x_n\right)$ be a polynomial function, where $x_i(1\le i\le n)$ represents the outsourced data. Reference^11^ gives the definition of the polynomial function $f\left( x_1,\ldots ,x_n\right) =\sum _{i=1}^{n}{(c_i*\prod _{j} x_j^{e_j})}$. Then drawing on the idea of Horner’s method^38^, we further represent the delegate function as Eq. (3).

3 $$\begin{aligned} &f\left( x_1,\ldots ,x_n\right) \\&=c_0+c_1\left( x_0^{e_{10}}x_1^{e_{11}}\ldots x_n^{e_{1n}}\right) \ldots c_n\left( x_0^{e_{n0}}x_1^{e_{n1}}\ldots x_n^{e_{nn}}\right) \\&=c_0+x_0\left( c_1x_0^{e_{10}-1}+\ldots +c_nx_0^{e_{n0}-1}\right) +\ldots \\ \end{aligned}$$

where $c_i$ represents the constant coefficient and $e_j$ represents the exponent of $x_j$. It requires that multiplication gates and addition gates can be interleaved to express the delegated polynomial function as shown in Fig. 2, which improves the situation that the addition gate must be carried out after the multiplication gate in the scheme^11^, so as to improve the verification efficiency. The cloud runs the polynomial function using the arithmetic circuit.

If the gate is a multiplication gate, then

1. The inputs are the constant $c\in Z_p$ and the variable *x* which has the verification tag $\delta (pk,\sigma \left( r,s\right) )$. For $y=x*c$, the GateVal() algorithm outputs the verification tag $\delta \prime (pk\prime ,\sigma \prime )$ as Eq. (4).
  4 $$\begin{aligned} pk^\prime&=pk=(g^a,h^a,h^\frac{1}{a}),t'=t_\tau c\\ \sigma ^\prime&=\left( r^\prime ,s\prime \right) =(r^c,s^c)=({(h}^{kc},h^{a\left( t_\tau c+xc+kc\right) }) \end{aligned}$$
2. The inputs are variable $x_1$ and $x_2$ with the verification tag $\delta _1({pk}_1,\sigma _1(r_1=h^{k_1},s_1=h^{a_1\left( t_{\tau 1}+x_1+k_1\right) }mod\ p))$ and $\delta _2({pk}_2,\sigma _2(r_2=h^{k_2},s_2=h^{a_2\left( t_{\tau 2}+x_2+k_2\right) }mod\ p))$ respectively. For $y=x_1*x_2$, the GateVal() outputs the verification tag $\delta \prime (pk\prime ,\sigma \prime )$.
  - The cloud sends $\sigma _2,x_2$ to KMC.
  - KMC verifies the signature $\sigma _2$ as shown in Eq. (2). If that fails, output $\bot$; otherwise, KMC randomly selects $k_2^\prime$, and uses the $a_1^\prime$ to generate $s_2^\prime =a_1^\prime \left( t_{\tau 2}+x_2+k_2^\prime \right)$, ${{\hat{r}=r}_1}^{t_{\tau 2}+x_2+k_2^\prime }$, $h^{k_2^\prime }$, $pk^\prime =(g^{a^*},h^{a^*},h^\frac{1}{a^*})$ , where $a_1 *a_1'=a^*$, and send them to the cloud.
  - The cloud computes verification tag $\sigma \prime =(r^\prime ,s^\prime )$.
    5 $$\begin{aligned} pk^\prime&=(g^{a^*},h^{a^*},h^\frac{1}{a^*}),{t\prime =t}_{\tau 1}t_{\tau 2} \\ \sigma ^\prime&=\left( r^\prime ,s^\prime \right) \\&=(\hat{r}*(h^{k_2^\prime })^{t_{\tau 1}+x_1}*h^{t_{\tau 1}x_2+x_1t_{\tau 2}},{s_1}^{s_2^\prime })\\&=((\hat{r}*(h^{k_2^\prime })^{t_{\tau 1}+x_1}*h^{t_{\tau 1}x_2+x_1t_{\tau 2}},{(h^{a_1(t_{\tau 1}+x_1+k_1)})}^{a_1^\prime (t_{\tau 2}+x_2+k_2^\prime )}))\\&=(h^\phi ,h^{a^*\left( t_{\tau 1}t_{\tau 2}+x_1x_2+\phi \right) }) \end{aligned}$$
    where $$\phi =t_{\tau 1}x_2+t_{\tau 1}k_2^\prime +x_1t_{\tau 2}+x_1k_2^\prime +k_1t_{\tau 2}+k_1x_2+k_1k_2^\prime$$.

If the gate is an additive gate ’+’, then

1. The inputs are the constant $c\in Z_p$ and the variable *x* which has the verification tag $\delta (pk,\sigma \left( r,s\right) )$. For $y=x+c$, the GateVal() algorithm outputs the verification tag $\delta \prime (pk\prime ,\sigma \prime )$ as Eq. (6).
  6 $$\begin{aligned} pk^\prime&=pk=(g^a,h^a,h^\frac{1}{a}),t\prime =t_\tau +H(c)\\ \sigma ^\prime&=\left( r^\prime ,s\prime \right) \\&=(r^k,s*h^{a(H(c)+c)})\\&={(h}^k,h^{a\left( (t_\tau +H(c))+(m+c)+k\right) }) \end{aligned}$$
2. The inputs are variable $x_1$ and $x_2$ with the verification tag $\delta _1({pk}_1,\sigma _1\left( r_1,s_1\right) )$ and $\delta _2({pk}_2,\sigma _2\left( r_2,s_2\right) )$ respectively. For $y=x_1+x_2$, the GateVal() algorithm outputs the verification tag $\delta \prime (pk\prime ,\sigma \prime )$.
  - If ${pk}_1={pk}_2$, this means that $\delta _1$ and $\delta _2$ have the same private key, i.e $a=a_1=a_2$.
    7 $$\begin{aligned} pk^\prime&=pk=(g^a,h^a,h^\frac{1}{a}),t^\prime =t_{\tau 1}+t_{\tau 2}\\ \sigma ^\prime&=\left( r^\prime ,s^\prime \right) \\&=\left( r_1*r_2,s_1*s_2\right) \\&=(h^{k_1+k_2},h^{a\left( t_{\tau 1}+t_{\tau 2}+(x_1+x_2)+k_1+k_2\right) }) \end{aligned}$$
  - If ${pk}_1\ne {pk}_2$, this means that $\delta _1$ and $\delta _2$ have different private keys, i.e $a_1\ne a_2$.

(i) The cloud sends $\sigma _1,\sigma _2$ to KMC.
(ii) KMC generates $s_1^\prime ={(s_1)}^{a_1^\prime }=h^{a^*\left( t_{\tau 1}+x_1+k_1\right) }$ using $a_1^\prime$, generates $s_2^\prime ={(s_2)}^{a_2^\prime }=h^{a^*\left( t_{\tau 2}+x_2+k_2\right) }$ using $a_2^\prime$, and send them to the cloud.
(iii) The cloud computes verification tag $\sigma \prime =(r^\prime ,s^\prime )$.
  8 $$\begin{aligned} pk^\prime&=(g^{a^*},h^{a^*},h^\frac{1}{a}),t^\prime =t_{\tau 1}+t_{\tau 2}\\ \sigma ^\prime&=\left( r^\prime ,s^\prime \right) \\&=\left( r_1*r_2,s_1^\prime *s_2^\prime \right) \\&=(h^{k_1+k_2},h^{a^*\left( t_{\tau 1}+t_{\tau 2}+(x_1+x_2)+k_1+k_2\right) }) \end{aligned}$$

#### $ProofCre\left( \delta \right) \rightarrow (P)$

The algorithm is executed by the cloud to generate the final proof message. The input is the verification tag by running the GateVal() on the last gate and the output is the final proof message $P=\delta _R(pk,\sigma )$. The cloud sends the proof message P to KMC.

#### $VerifyProof\left( P\right) \rightarrow (True,False)$

The algorithm is executed by KMC to verify the results of the polynomial calculations. The input is proof message P, and the output is True or False. True shows that the result is correct, False shows that the result is incorrect.

KMC receives the calculation result of the function $R=f\left( x_1,\ldots ,x_n\right)$ and the proof information $P=\delta _R(pk,\sigma (r,s))$. Given that each input $x_i$ of the polynomial has a label $\tau _i$, KMC computes $t_i=H(\tau _i)$, then KMC computes $\rho \leftarrow f\left( t_1,\ldots ,t_n\right)$. The correctness of the result *R* is verified using *P*. If the check is passed, the result *R* is correct and the output is True. Otherwise, the result *R* is incorrect and the output is False.

9 $$\begin{aligned} e\left( g,s\right) =e({pk}^{\left( 1\right) },r*h^\rho *h^R) \end{aligned}$$

In practice, the data $\rho \leftarrow f\left( t_1,\ldots ,t_n\right)$ can be generated and stored in advance to increase efficiency.

### Security analysis

We analyzed the security of the scheme from two aspects: correctness and soundness. First of all, we confirm that the verification tag designed in this scheme support addition homomorphism and multiplication homomorphism, and on this basis we verify the correctness of the scheme based on the Computational Diffie-Hellman (CDH) Assumption. Secondly, we confirm the soundness of the scheme, in which the verification tag forged by the attacker cannot pass the verification test.

### Correctness

We verify that the verification tag designed by the scheme support addition homomorphism and multiplication homomorphism, and then we verify the correctness of the scheme based on CDH hypothesis.

#### Lemma 1

*The verification tag is additive homomorphic.*

#### Proof

 In the addition gate, the inputs $x_1$ and $x_2$ have the labels $\tau _1$ and $\tau _2$ (for the constant *c*, the labels are *c*), and get $t_{\tau 1}=H(\tau _1)and t_{\tau 2}=H(\tau _2)$. For $y=x_1+x_2$, the cloud generates verification tags $\sigma ^\prime =\left( r^\prime ,s^\prime \right) =(h^{k_1+k_2}$, $h^{a^*\left( t_{\tau 1}+t_{\tau 2}+(x_1+x_2)+k_1+k_2\right) })$, where $a^*$ is the security parameter selected by KMC. Therefore, KMC can verify the correctness of $y=x_1+x_2$ by Eq. (10) using the verification tags without knowing $x_1$ and $x_2$.

10 $$\begin{aligned} e\left( g,s\right)&=e(g^{a^*},h^{a^*\left( {(t}_{\tau 1}+t_{\tau 2})+(x_1+x_2)+{(k}_1+k_2)\right) })\\&=e({pk}^{\left( 1\right) },r\prime *h^{t_{\tau 1}+t_{\tau 2}}*h^{x_1+x_2}) \end{aligned}$$

It is obvious that verification tags are additive homomorphic.

#### Lemma 2

* The verifying tag is multiplicative homomorphic*.

#### Proof

 In the multiplication gate, the inputs $x_1$ and $x_2$ have the labels $\tau _1$ and $\tau _2$ (for the constant *c*, the labels are *c*), and get $t_{\tau 1}=H(\tau _1)$ and $t_{\tau 2}=H(\tau _2)$. For $y=x_1*x_2$, the cloud generates verification tags $\sigma ^\prime =\left( r^\prime ,s^\prime \right) =(h^k,h^{a^*\left( t_{\tau 1}t_{\tau 2}+x_1x_2+k\right) })$, where $a^*$ is the security parameter selected by KMC. Therefore, KMC can verify the correctness of $y=x_1*x_2$ by Eq. (11) using the verification tags without knowing $x_1$ and $x_2$.

11 $$\begin{aligned} e\left( g,s\right)&=e(g^{a^*},h^{t_{\tau 1}t_{\tau 2}+x_1x_2+k})\\&=e({pk}^{\left( 1\right) },r\prime *h^{t_{\tau 1}t_{\tau 2}}*h^{x_1x_2}) \end{aligned}$$

It is obvious that verification tags are multiplicative homomorphic.

#### Theorem 1

*The correctness of the scheme is achieved.*

#### Proof

 According to Lemmas 1 and 2, the verification tag of this scheme is a homomorphic verifiable label. KMC can verify the correctness of the calculation results without knowing the input. The correctness of this scheme is equivalent to proofing the correctness of VerifyProof(). The correctness of Eq. (9) can be verified by Eq. (12).

12 $$\begin{aligned} e\left( g,s\right)&=e(g,h^{a(\rho +R+k)})\\&=e\left( g^a,h^{\rho +R+k}\right) \\&=e({pk}^{\left( 1\right) },r*h^\rho *h^R) \end{aligned}$$

### Soundness

#### Theorem 2

* The soundness of the scheme is achieved*.

#### Proof

We demonstrate the soundness of the scheme, which shows that once the cloud or external attacker is able to pass the verification by forging the verification tag with false result, they are able to establish adversary $\mathcal {A}$ with a non-negligible probability.

Assume that the security parameters of the system are $(e,p,G_1,G_2,h,H)$, where $h=g^x,x\in Z_p$. Define the proof information $P=\delta _R(PK,\sigma (r,s)), {PK}^{(1)}=g^a,$ where $a=x*y(y\in Z_p)$. The adversary $\mathcal {A}$ outputs CDH challenge as $(g^{xy},g^y)=({PK}^{(1)},h)$. Define $q_{H_i}$ as the number of times to count the process $t_\tau =H\left( \tau \right)$. Thus, the probability of the collision occurring in *H* used to compute $t_{\tau _i}$ is at most $q_{H_i}/2^l$, where *l* is the length of the output of *H*. Based on Reset Lemma^39^, adversary $\mathcal {A}$ can generate two verification tags $\delta _1(PK,\sigma _1\left( r,s_1\right) ),\ \delta _2(PK,\sigma _2\left( r,s_2\right) )$ with the possibility at least $\prod _{i=1}^{n}{(\epsilon -\left( \epsilon *q_{H_i}+1\right) /2^l)}^2$.

The adversary $\mathcal {A}$ forge verification tags $\sigma _1(r=h^k,s_1=h^{a\left( \rho _1+R+k\right) }mod\ p),\sigma _2(r=h^k,s_2=h^{a\left( \rho _2+R+k\right) }mod\ p)$, where $\rho _1,\rho _2$ are two different outputs of $\rho \leftarrow f\left( t_1,\ldots ,t_n\right)$. Adversary $\mathcal {A}$ can solve the CDH problem by calculating:

$$\begin{aligned} {(\frac{s_1}{s_2})}^\frac{1}{(\rho _1-\rho _2)} ={(\frac{h^{a\left( \rho _1+R+k\right) }}{h^{a\left( \rho _2+R+k\right) }})}^\frac{1}{(\rho _1-\rho _2)}=h^a={(g^x)}^{xy}=g^{x^2y} \end{aligned}$$

It is obvious that if the attacker can forge the verification tag, then we can solve the CDH problem, which is impossible. Therefore, the verifiable computation scheme is soundness, that is, attacker cannot fabricate proof information for any wrong result.

## Performance analysis

### Communication cost

In the polynomial verifiable computation scheme, there are two types of communication costs.

1. The cloud needs to communicate with KMC to transmit proof information. The proof information is expressed as $P=\delta _R(pk,\sigma )$, so the communication cost of the proof information is $\left| S_{pk}\right| +\left| S_\sigma \right|$, where the size of the public key *pk* is $\left| S_{pk}\right|$ and the size of the signature $\sigma$ is $\left| S_\sigma \right|$.
2. The cloud needs to communicate with KMC to generate corresponding security parameters in GateVal() algorithm. The communication cost of the generated intermediate parameter in the multiplication gate is $$c_**\left| S_G\right|$$, where $$\left| S_G\right|$$ indicates the size of the data in $$G_1$$, and $$c_*$$ is 0 or 1 indicates the two calculation methods of the multiplication gates. For the additive gate of the polynomial function, the communication cost of the generated intermediate parameter is $$c_+*\left| S_G\right|$$, where $$c_+$$ is 0, 1, and 2 indicate the three computation methods of the additive gates. Thus the communication cost is $$\left( c_**{sum}_*\right) *\left| S_G\right| +{(c}_+*{sum}_+)*\left| S_G\right| ={(c}_+*{sum}_++c_**{sum}_*)*\left| S_G\right|$$, where $${sum}_*$$ represents the number of multiplication gates in the circuit and $${sum}_+$$ represents the number of multiplication gates in the circuit.

### Computation cost

We assume $T_{exp},T_{add},T_{mul},T_{hash},T_{mod},T_{pair}$ represent exponentiation operation, addition operation, multiplication operation, hash operation, module operation, and pairing operation of bilinear mapping respectively.

The calculating cost of Sign() is $2T_{exp}$.

In the GateVal(), the calculating cost is $2T_{exp}$ for Eq. (4), the calculating cost is ${2T}_{add}+3T_{mul}+2T_{hash}+3T_{exp}$ for Eq. (5), the calculating cost is $2T_{exp}+T_{add}+2T_{mul}+T_{hash}$ for Eq. (6), the calculating cost is ${2T}_{exp}$ for Eq. (7), the calculating cost is ${2T}_{mul}$ for Eq. (8).

The calculating cost of ProofGen() is the sum of the calculating costs of all gates $\sum _{g\in \left| f\right| } T_g$, where $\left| f\right|$ represents the set of gates in a polynomial function, and $T_g$ represents the calculating cost of performing an addition or multiplication gate of the GateVal() algorithm.

The calculating cost of VerifyProof() is ${2T}_{exp}+2T_{mul}+T_{pair}+T_\rho$, where $T_\rho$ indicates the calculating cost of computing $\rho \leftarrow f\left( t_1,\ldots ,t_n\right)$.

We compare the proposed scheme in the paper with existing solutions^11^, as shown in Table 3. In Table 3, we can see that their calculating costs are similar during the signature stage. However, in practice, the signature method of the two schemes are different in design and operation. There are two signature methods designed in the existing scheme^11^, which may be due to the requirement that the addition gate must be executed after the multiplication gate when generating the verification tag. This approach affects the generation efficiency of verification tag in subsequent steps. In contrast, the proposed scheme in the paper does not have this limitation, so it can generate verification tag more efficiently. In addition, the scheme in the paper uses Horner’s method to optimize polynomials when generating verification labels. We can see that the GateVal() and ProofCre() algorithms are used to generate verification tag, which is more efficient than existing scheme^11^. This method can make the generation of verification tag faster, thereby improving the efficiency of the entire scheme. In contrast, the existing schemes^11^ may not adopt this optimization method, resulting in slower generation of verification tag. We can find that the difference in calculating cost between the two schemes mainly exists when the inputs are the variable $x_1,x_2$ with the different public key. This is because for the polynomial-based verifiable computation scheme on multi-source outsourced data, the design of this part is the difficulty and focus. The scheme^11^ require that addition gates must be executed after multiplication gates, and design complex two-level tags, resulting in inefficiency. The proposed scheme improves this by using a unified verification tag, allowing multiplication and addition gates to be executed in parallel, resulting in improved efficiency of verification tag generation.

To sum up, we use Horner’s method to optimize the polynomial, which will make the system execute the addition gate or multiplication gate significantly less times than the existing scheme. This is our main idea to improve efficiency. In order to adapt to this structure, an efficient verification tag is designed to support addition homomorphism and multiplication homomorphism, so that the addition gate and multiplication gate can be crossed and the verification speed can be further improved.As a result, the proposed scheme on efficiency is better than the existing scheme.

**Table 3:** Comparison with scheme^11^.

Scheme	The scheme^11^	The proposed scheme	The input of algorithm
*Sign*()	$T_{exp}+{2T}_{add}+T_{mul}+T_{hash}+T_{mod}$	$T_{exp}+{2T}_{add}+T_{mul}+T_{hash}+T_{mod}$	The inputs is the outsourced data *x*
multiplication gate in GateVal()	$T_{exp}+T_{mul}$	$2T_{exp}$	The inputs are the constant $c\in Z_p$ and the variable *x*
	${2T}_{add}+4T_{mul}+2T_{hash}+2T_{exp}$	${2T}_{add}+3T_{mul}+2T_{hash}+3T_{exp}$	The inputs are the variable $x_1,x_2$
additive gate in GateVal()	${2T}_{exp}$	${2T}_{exp}$	The inputs are the variable $x_1,x_2$ with the same public key
	$2T_{exp}+T_{add}+2T_{mul}+T_{hash}$	$2T_{exp}+T_{add}+2T_{mul}+T_{hash}$	The inputs are the constant $c\in Z_p$ and the variable *x*
	${2T}_{exp}+2T_{mul}$, where the verification tags of $x_1,x_2$ are both 1-level verification tag; $T_{exp}+T_{add}+T_{mul}+T_{hash}$, where the inputs are a constant $c\in Z_p$ and the variable *x* with the 2-level verification tag; ${2T}_{mul}+T_{exp}$, where the verification tag of $x_1$ is 1-level verification tag, the verification tag of $x_2$ is 2-level verification tag; ${2T}_{mul}+T_{exp}$, where the verification tags of $x_1,x_2$ are both 2-level verification tag	${2T}_{mul}$	The inputs are the variable $x_1,x_2$ with the different public key
ProofCre()	$\sum _{g\in \left| f\right| } T_g$	$\sum _{g\in \left| f\right| } T_g$	The verification tag
VerifyProof()	${2T}_{exp}+2T_{mul}+T_{pair}+T_\rho$	${2T}_{exp}+2T_{mul}+T_{pair}+T_\rho$	The final proof message

## Experimental results

### Experiment setup

The experiment was conducted in the environment of Intel(R) Core(TM) i5-10210U CPU @ 1.60 GHz 2.11 GHz. The dataset we used was air pollution data for 367 major cities in China. In the experiment, we uploaded air pollution data as raw data to cloud server. This air pollution data will be used for data analysis, and the results of the data analysis will be published. These data analyses will be used to calculate the average air quality of all cities in the country, the average value of a certain pollution component in a city, etc.

The performance of the scheme is compared with homomorphic MAC^10^, ADSNARK^40^, and verifying tag^11^. The homomorphic MAC^10^ does not support multiple data sources. We mainly measure the efficiency of the scheme from three aspects: the calculating cost of signature generation, the calculating cost of proof information generation, the calculating cost of verification phase, etc.

### The calculating cost of signature generation

**Figure 3:**
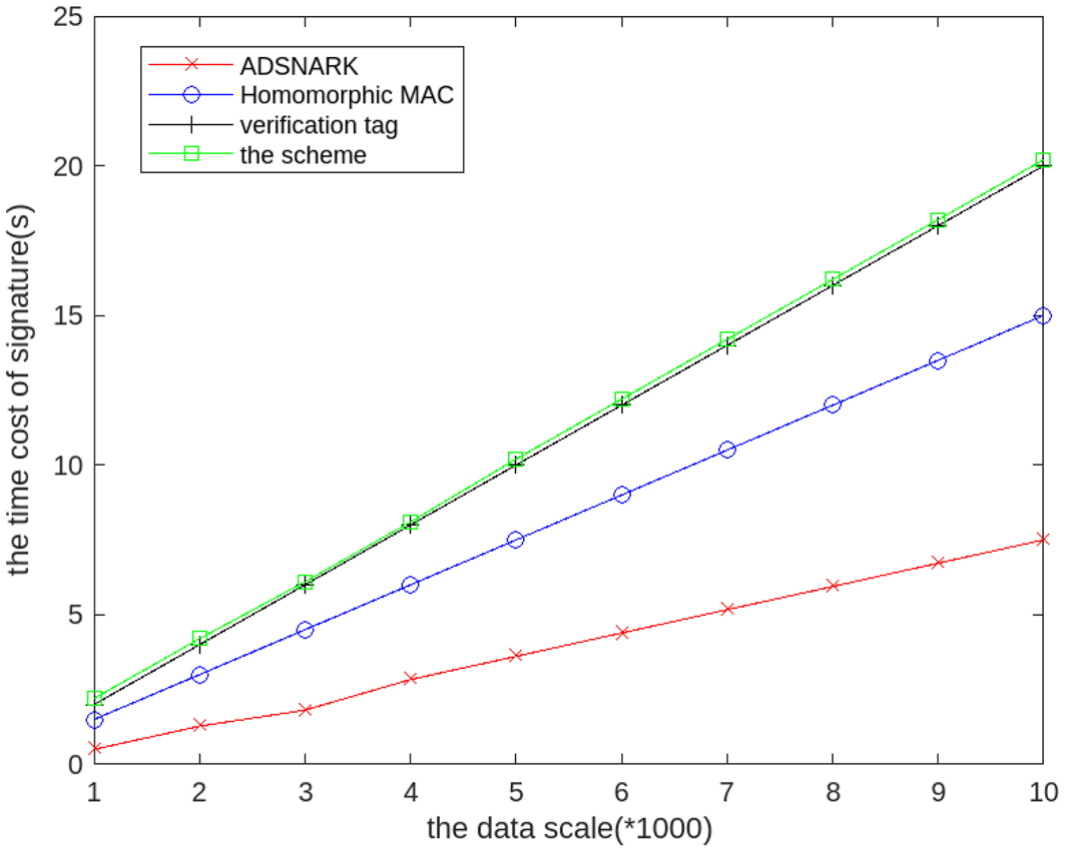
The calculating cost of the signature generation with different data scale.

In the signature generation, the data is signed and uploaded to the cloud along with the original data. We must ensure the efficiency of signing, which is closely related to the efficiency of the offline phase. In order to verify the calculating cost of the signature generation algorithm Sign(), we compare the calculating cost of the Sign() algorithm in the scheme, the homomorphism MAC^10^ , the ADSNARK^40^ and verifying tag^11^, where the data scale ranges from 1000 to 10,000. As shown in the Fig. 3, the calculating cost of signature increases with the increase of data volume. The time cost of signature generation in this scheme is similar to verifying tag^11^. Specially, the signature generation can be generated offline at the user side before the data is uploaded to the cloud, which will not affect the data correctness checks at the online stage. And Homomorphic MAC cannot directly support multiple data contributors, so this scheme can generate signatures relatively quickly and the input of polynomials support multiple data sources.

### The calculating cost of proof information generation

After the signature is generated, the cloud generates the proof information by algorithm ProofGen(). Here we evaluate the calculating cost of the algorithm ProofGen() from two perspectives.

First, one of the important motivations of our scheme is to improve efficiency and to be suitable for polynomial calculations of higher order. So we evaluate the calculating cost of polynomial functions with different order sizes. In order to comprehensively measure the efficiency of the scheme, we randomly select some polynomials with high order, where the order of polynomial function range from 50 to 500. We compare the ProofGen() algorithm in the scheme, the homomorphism MAC , the ADSNARK and verifying tag, and we assume that all data is outsourced and signed by a single user.

**Figure 4:**
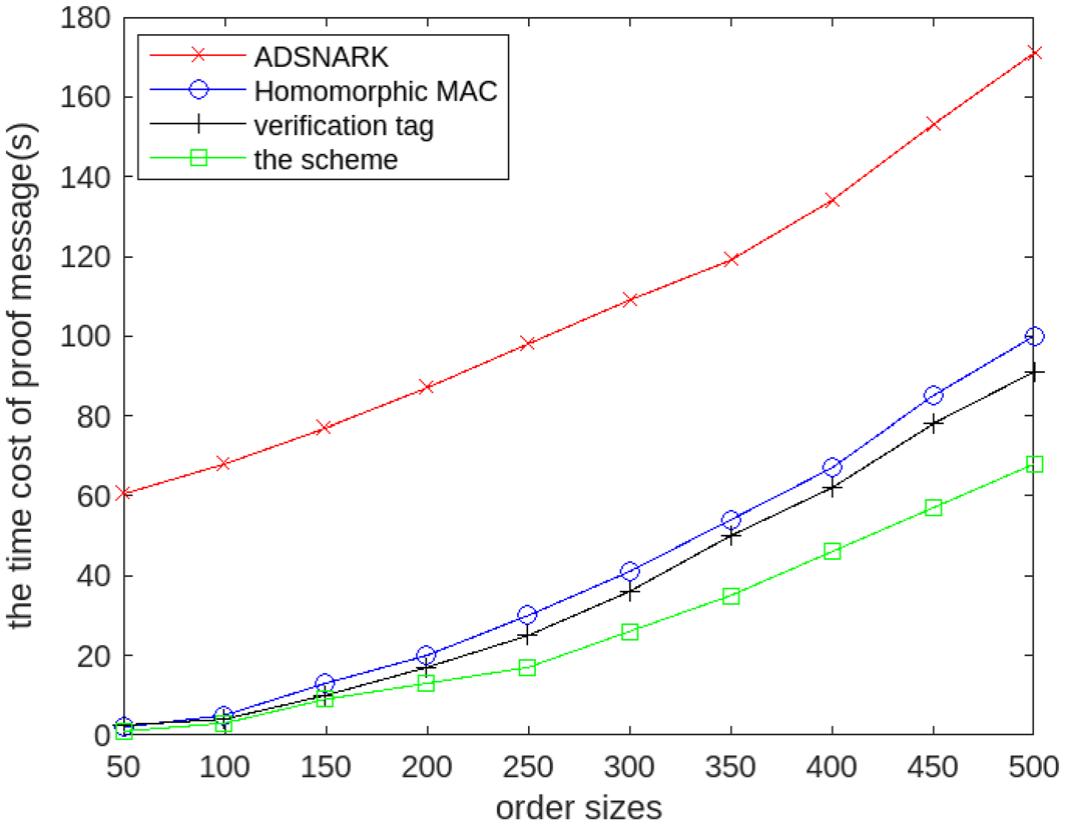
The calculating cost of the proof information generation with different polynomial order.

Figure 4 illustrates the calculating cost of the scheme will not increase obviously with the increase of polynomial order. And this scheme consumes less time compared with the other schemes^10,11,40^, which shows this scheme can effectively reduce the calculating cost of proof information generation. In particular, the scheme can generate verification tag much faster when the order of polynomials is high. This is because the scheme is not limited by the polynomial structure, and the multiplicative gate and the additive gates can be executed interactively. When polynomials are optimized by Horner’s Method, the verification tag generation can be faster compared to other schemes.

Second, one of the motivations for our scheme is that it is suitable for cases where the input of a polynomial comes from multiple data sources. So we evaluate the calculating cost of polynomial functions with different number of data owners, where the number of data owners ranges from 1 to 300. In the experiment, the input of the polynomial is pollution data from multiple cities, which will be signed using different private keys. The homomorphic MAC cannot directly support multiple data contributors, so we compare the ProofGen() algorithm in the scheme, the ADSNARK and verifying tag.

**Figure 5:**
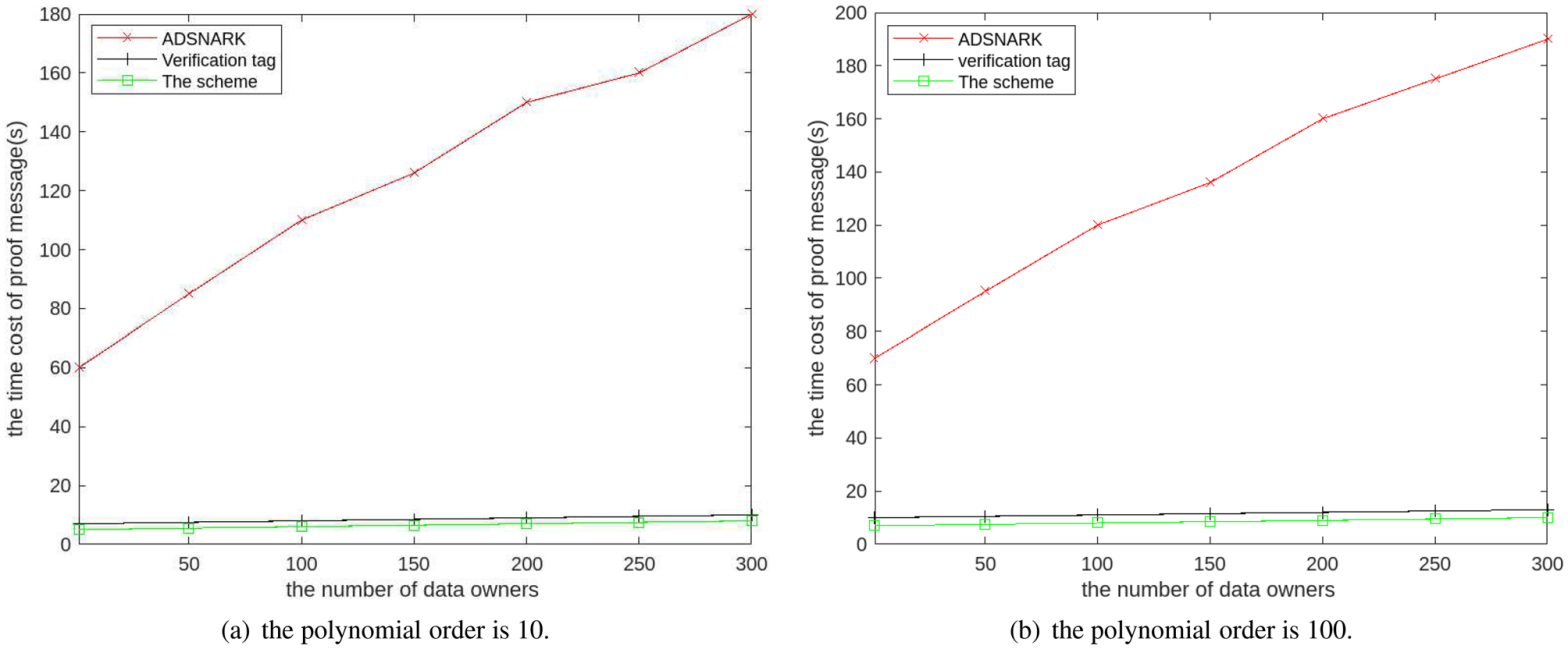
The calculating cost of proof information generation in the cloud with different number of data owner.

Figure 5 illustrates the algorithm ProofGen() is executed very quickly on the cloud server and does not significantly increase even the data sourcing from multiple users. It is very obvious that the scheme can perform calculations much faster than the other schemes. By comparing two subgraphs, the time cost does not increase significantly with the increase of polynomial order, which indicates that the scheme is suitable for complex polynomials. Therefore, this scheme can verify the correctness of the calculated results very quickly, in which the data can be derived from multiple data sources.

### The calculating cost of verification phase

After the proof information is sent to the Key management center, the Key management center executes the algorithm VerifyProof() to verify the accuracy of the calculation results using the proof information. We evaluate the calculating cost of verification phase with different order sizes of polynomial function, and we assume that all data is outsourced and signed by a single user. We compare the ProofGen() algorithm in the scheme, the homomorphism MAC , the ADSNARK and verifying tag, and we assume that all data is outsourced and signed by a single user.

**Figure 6:**
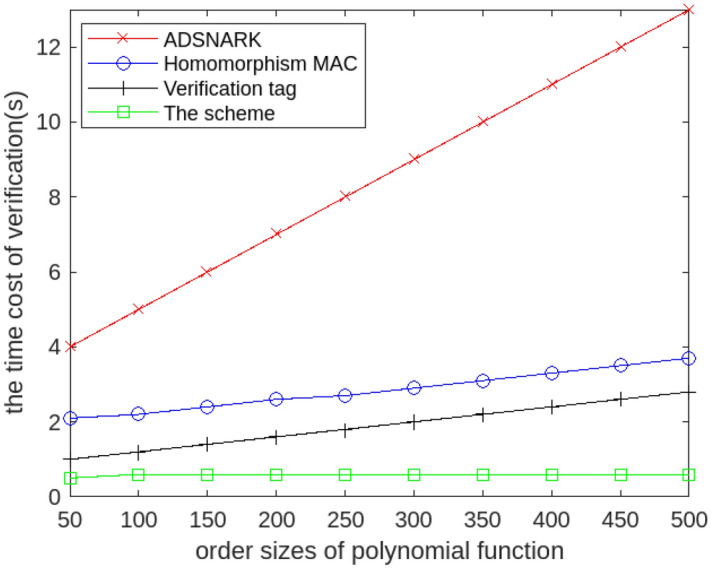
The calculating cost of the verification phase with different polynomial order.

Figure 6 illustrates that the calculating cost of the scheme does not increase significantly with the increase of polynomial order. This is because even if the data comes from a large number of users, only one proof information corresponding to one result can be generated after polynomial calculation. Therefore, the time consumption during the verification phase does not significantly increase due to the increase in data sources. Then it turns out that the calculating cost of the scheme is smaller than that of other schemes. The experimental results show that the proposed scheme can quickly and effectively verify the correctness of the calculated results, even if the data comes from multiple data sources.

## Discussion

This paper presents an efficient polynomial-based verifiable computation scheme for multi-source outsourced data. We optimize polynomials for faster verification using the Horner method, where addition and multiplication gates can interlace polynomial functions. In order to adapt to this structure, we design the corresponding homomorphic verification labels, so that the input of the polynomial can come from multiple data sources. Our proposal has some important advantages. First, it works with multi-source data, which means that the values of the input polynomials can come from multiple users. This can be important in practical applications, such as in distributed systems or secure multi-party computing. Secondly, our solution is efficient. By using the Horner method, we can reduce the amount of computation required, which speeds up verification.

However, our proposal also has some potential limitations and directions for future research. First, our scheme is only suitable for verifying the correctness of the calculation results of entrusted polynomial functions, and may not be suitable for all types of data and computation tasks. Then, the work of user in the preprocessing stage may be complex, and it will consume a certain amount of computing resources and storage resources in user side. To solve these problems, the future work will focus on extending this scheme to handle more complex polynomial functions and to further enhance its efficiency. We also plan to investigate the application of this scheme in other fields, such as cryptography and distributed computing, where polynomial-based computations play a crucial role. Additionally, we aim to develop more secure and privacy-preserving methods for outsourced data computation to address the concerns of untrustworthy cloud servers.

## Conclusion

Verifiable computing means that the computing task is outsourced to the untrusted cloud server, and the untrusted cloud server needs to submit a correctness proof of the calculation results while completing the computing task. There are two main problems with the existing verifiable computing scheme. First, the existing scheme requires that the input of the polynomial must come from a single data source. Secondly, the design of verification labels may cause problems such as reduced efficiency, especially when the polynomial function is relatively complex, so that the verification process will be extremely slow, and even affect the use of data. To solve these problems, we design for the first time an efficient polynomial-based verifiable computation scheme on multi-source outsourced data, which has the characteristics of efficient and supporting multiple data sources. As the polynomial function is executed gate by gate, we use the key management center to convert the signatures signed by different user into the verification tag with the unified public and private keys, so that the input of the polynomial can come from multiple data sources. Specially, we optimize the polynomials using Horner’s Method, and the generation of corresponding verification tag can be generated with the cross-operator of multiplication gate and addition gate, so as to improve the efficiency. Then we demonstrate the security of the scheme from two aspects: correctness and soundness. The performance of the scheme is verified by experiments, which shows that the scheme is more efficient than the existing schemes. Therefore, the scheme is able to provide efficient verifiable computing services in cloud outsourcing services, where the input of polynomials can come from multiple data sources. Overall, the work presented in this paper represents a significant step forward in achieving efficient and secure verifiable computation on multi-source outsourced data. We believe that our future research will further enhance the capabilities and applicability of this scheme, paving the way for more reliable and privacy-preserving cloud computing services.

## Data Availability

The data used in the experiment can be downloaded at http://www.cnemc.cn/. Moreover, more data used and analysed during the current study available from the corresponding author on reasonable request.
